# A121 ENDOSCOPIC RETROGRADE CHOLANGIOPANCREATOGRAPHY IN PATIENTS WITH PANCREATICODUODENECTOMY USING DYNAMIC RIGIDIZING OVERTUBE

**DOI:** 10.1093/jcag/gwac036.121

**Published:** 2023-03-07

**Authors:** D Low, C Liu, J Lee, F Shaukat Ali, A Al Nakshabandi, E Coronel

**Affiliations:** MD Anderson Cancer Center, Houston, United States

## Abstract

**Background:**

Dynamic rigidizing overtubes have recently been described as a useful tool in challenging endoscopic procedures. These devices have the ability to dynamically rigidize, which stabilizes endoscope position, reduce loop formation and assist in the traversal of acute angulation. Its use has been best described in difficult colonoscopies by preventing loop formation and subsequently facilitating cecal intubation. However, the use of rigidizing overtubes for endoscopic retrograde cholangiopancreatography is sparsely described in the literature. In particular, rigidizing overtubes may be beneficial in patients with surgically-altered anatomy, including post-pancreaticoduodenectomy (PD) patients, as traversal to the surgical anastomosis is often challenging because of loop formation and acute angulation. In this study, a retrospective analysis was conducted on post-PD patients undergoing ERCP with dynamic rigidizing overtube.

**Purpose:**

The purpose of this study was to evaluate the technical and clinical success of post-PD patients undergoing ERCP with rigidizing overtube.

**Method:**

A retrospective analysis was conducted on all post-PD patients who underwent ERCP with rigidizing overtube between 2006 and 2021. Demographic information including age, gender and primary malignancy was collected. Pertinent procedure-related information including indication and anastomosis identification was obtained. The primary outcomes evaluated included technical and clinical success rate. Technical success (TS) was defined as the ability to perform an intended therapeutic intervention (stent insertion, cannulation, etc). Clinical success (CS) was operationalized as improvement in patient symptoms or normalization of bilirubin.

**Result(s):**

A total of 5 patients underwent 11 ERCPs with rigidizing overtube for biliary indications. There was a total of 3 (60.0%) male and 2 (40.0%) female patients. The average age of the patients was 62.5 years of age. There were 3 (60.0%) patients with pancreas cancer, 1 (20.0%) with metastatic renal cell carcinoma, and 1 (20.0%) with duodenal adenoma with high grade dysplasia. The indications for ERCP included stent evaluation (n = 6; 54.5%), obstructive jaundice (n =3; 27.3%), cholangitis (n = 1; 9.1%), and stricture evaluation (n = 1; 9.1%). The surgical anastomosis was identified in all patients (n = 11; 100%). The overall technical and clinical success rates were both 90.9%.

**Conclusion(s):**

The use of rigidizing overtubes in ERCP may be beneficial in patients with surgically altered anatomy. In this small sample, technical and clinical success rates were excellent in post-PD patients. However, additional studies need to be conducted to validate these findings further.

**Please acknowledge all funding agencies by checking the applicable boxes below:**

None

**Disclosure of Interest:**

None Declared

